# Predicting MGMT Promoter Methylation in Diffuse Gliomas Using Deep Learning with Radiomics

**DOI:** 10.3390/jcm11123445

**Published:** 2022-06-15

**Authors:** Sixuan Chen, Yue Xu, Meiping Ye, Yang Li, Yu Sun, Jiawei Liang, Jiaming Lu, Zhengge Wang, Zhengyang Zhu, Xin Zhang, Bing Zhang

**Affiliations:** 1 Department of Radiology, The Affiliated Drum Tower Hospital of Nanjing University Medical School, Nanjing 210008, China; anzhitinglannju@foxmail.com (S.C.); yemeipingmm@163.com (M.Y.); licheng1573@foxmail.com (Y.L.); vector_6@163.com (J.L.); secwang235@163.com (Z.W.); 161230042@smail.nju.edu.cn (Z.Z.); zhangbing_nanjing@nju.edu.cn (B.Z.); 2 National Institute of Healthcare Data Science, Nanjing University, Nanjing 210023, China; yue.xu5@mail.mcgill.ca; 3 School of Biological Science and Medical Engineering, Southeast University, Nanjing 211189, China; sunyu@seu.edu.cn (Y.S.); liangjiawei97@live.com (J.L.); 4 Institute of Brain Science, Nanjing University, Nanjing 210023, China

**Keywords:** MGMT promoter methylation, glioma, deep learning, radiomic

## Abstract

This study aimed to investigate the feasibility of predicting oxygen 6-methylguanine-DNA methyltransferase (MGMT) promoter methylation in diffuse gliomas by developing a deep learning approach using MRI radiomics. A total of 111 patients with diffuse gliomas participated in the retrospective study (56 patients with MGMT promoter methylation and 55 patients with MGMT promoter unmethylation). The radiomics features of the two regions of interest (ROI) (the whole tumor area and the tumor core area) for four sequences, including T1 weighted image (T1WI), T2 weighted image (T2WI), apparent diffusion coefficient (ADC) maps, and T1 contrast-enhanced (T1CE) MR images were extracted and jointly fed into the residual network. Then the deep learning method was developed and evaluated with a five-fold cross-validation, where in each fold, the dataset was randomly divided into training (80%) and validation (20%) cohorts. We compared the performance of all models using area under the curve (AUC) and average accuracy of validation cohorts and calculated the 10 most important features of the best model via a class activation map. Based on the ROI of the whole tumor, the predictive capacity of the T1CE and ADC model achieved the highest AUC value of 0.85. Based on the ROI of the tumor core, the T1CE and ADC model achieved the highest AUC value of 0.90. After comparison, the T1CE combined with the ADC model based on the ROI of the tumor core exhibited the best performance, with the highest average accuracy (0.91) and AUC (0.90) among all models. The deep learning method using MRI radiomics has excellent diagnostic performance with a high accuracy in predicting MGMT promoter methylation in diffuse gliomas.

## 1. Introduction

According to the 2021 WHO classification of tumors of the central nervous system (2021 WHO CNS), molecular classification is one of the most important prognostic factors. Oxygen 6-methylguanine-DNA methyltransferase (MGMT) promoter methylation is a significant prognostic factor in diffuse glioma patients, since diffuse glioma patients with MGMT methylation are shown to have a better prognosis and better response to temozolomide [1,2,3]. MGMT promoter methylation is related to longer progression-free survival in patients treated with radiochemotherapy or radiation alone [4]. The detection of MGMT promoter methylation plays an important role in the diagnosis of diffuse gliomas. At present, the detection of the MGMT promoter mainly depends on genetic analysis after a tumor operation or biopsy, which is expensive and requires invasive surgery [5].

Conventional magnetic resonance imaging (MRI) plays an important role in determining glioma patient diagnosis, prognosis, response evaluation, and follow-up. However, the status of MGMT promoter methylation is difficult to determine via conventional diagnosis of MR images. With the rapid development of molecular diagnosis and artificial intelligence, the study of tumor biomarkers by radiomics has become a hot research topic [6,7,8,9]. Radiomics generally refers to the extraction and analysis of a great number of advanced quantitative imaging features with high throughput from medical images [10,11]. These features, i.e., radiomics features, can reveal potential tissue and lesion characteristics, such as tumor heterogeneity. Radiomics has been widely used to predict molecular markers in gliomas, such as isocitrate dehydrogenase mutations [12,13,14]. Deep learning is a kind of machine learning method that attempts to model high-level abstractions in data by using multiple processing operations (layers), which has been effectively employed in solving image-based problems, including medical imaging [15,16,17,18]. Studies on the relationship between MGMT promoter methylation and MRI imaging of glioma are insufficient, and predicting the methylation status of the MGMT promoter in glioma with MR images is still a challenging task, requiring further research.

In this paper, a residual network (ResNet) was trained to give a binary prediction of MGMT promoter methylation status. Instead of using images as an input, as in existing research [19], our research extracted radiomics features from a selected region of interest (ROI) in different modalities of MR images and used them as the input of the model.

The purpose of this study is to develop a deep learning model following the hypothesis that radiomics combined with deep learning would be helpful for predicting the MGMT promoter status of diffuse gliomas. This work also has clinical significance that would help clinicians make appropriate treatment decisions.

## 2. Materials and Methods

### 2.1. Patient Cohort and Inclusion Criteria

**Figure 1:**
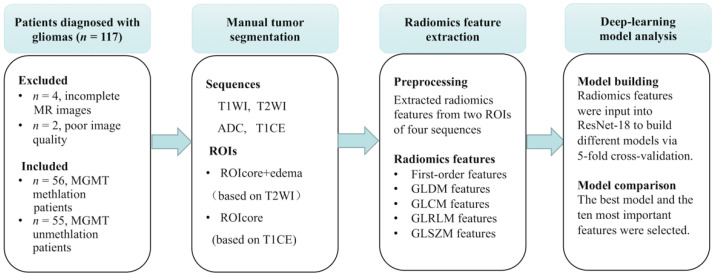
Workflow of our research. Radiomics features of 111 glioma patients were extracted from two regions of interest (the whole tumor area and the tumor core area) of four sequences, including T1 weighted image (T1WI), T2 weighted image (T2WI), apparent diffusion coefficient (ADC) maps, and T1 contrast-enhanced (T1CE) MR images were jointly fed into the ResNet-18. The performance of all models was compared using area under the curve (AUC) and average accuracy of validation cohorts. The 10 most important features of the best model were calculated via a class activation map. Abbreviations: oxygen 6-methylguanine-DNA methyltransferase (MGMT); residual network (ResNet); region of interest (ROI).

This retrospective study was approved by the local Institutional Review Board (IRB), and the requirement to obtain informed consent was waived. Patients were recruited from the Affiliated Drum Tower Hospital of Nanjing University Medical School between 2018 and 2020. The inclusion criteria for the study were as follows: surgical resection and pathology confirmed WHO grade 2–4 glioma according to the WHO 2021 version of the central nervous system tumor classification, and none of the patients received radiotherapy, chemotherapy or underwent antitumor drug treatment before surgery. Plain scan and enhanced examination were performed on the same MRI machine before surgery. Patients with incomplete images and poor image quality that could not be used for image analysis were excluded. Finally, a total of 111 patients meeting the above criteria were enrolled in the study, including 56 patients with MGMT promoter methylation and 55 patients with MGMT promoter unmethylation. The data were divided into a training group and a validation group at a ratio of 8:2, with 89 patients in the training group and 22 patients in the validation group. An overview of the workflow is shown in Figure 1.

### 2.2. MRI Data and Image Preprocessing

All MRI data analyzed in the present study were preoperatively acquired using 3.0 T MRI scanners according to the protocols in each institution, including the Philips instrument (Achieva TX, Philips Medical Systems, The Netherlands) and United imaging instrument (uMR770, United Imaging Healthcare, China), and the parameters are shown in Table S1. Available preoperative MRI images included T1 weighted image (T1WI), T2 weighted image (T2WI), diffusion-weighted imaging (DWI), apparent diffusion coefficient (ADC) maps, and T1 contrast-enhanced (T1CE) MR images. We eliminated the difference in MRI image brightness caused by the deviation in the scanning process and performed N4 deviation field correction on all MRI images.

### 2.3. Tumor Segmentation and Feature Extraction

**Figure 2:**
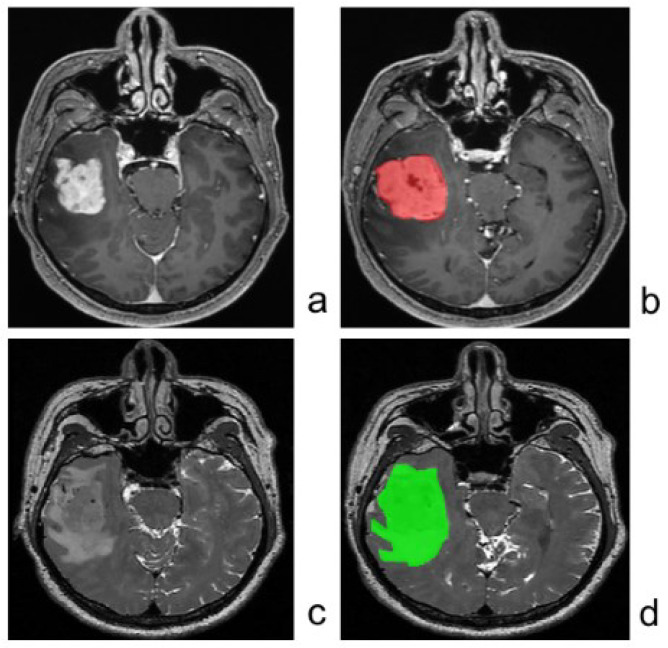
Process of delineating the ROI. The ROIs of the tumor parenchyma (red area) were delineated by manually tracing contrast-enhancing lesions on T1CE (**a**,**b**). The ROIs of whole tumors (green area), including the parenchyma and edema, were delineated by manually tracing high-intensity lesions on T2WI (**c**,**d**).

We extracted radiomics features from the tumor edema area and tumor core area of four sequences, including T1WI, T2WI, T1CE, and ADC. ROIs were easy to determine for tumors with clear boundaries; however, it was difficult to draw ROIs for gliomas with blurred boundaries and large edema areas. T2WI has great advantages in determining the ROI of edema areas, and T1CE is also very important in determining the ROI of the tumor parenchyma, which is the core region of the tumor. Therefore, the ROI of the whole tumor, including the parenchyma and edema, was manually segmented on T2WI, and the ROI of the tumor core was manually segmented on T1CE by two radiologists who were blinded to all clinical data and histopathological information (Figure 2). If the difference between the ROIs outlined by the two radiologists was less than 5%, the two ROIs were fused. If the difference between the two ROIs was greater than 5%, a third radiologist would make the final determination. Segmentation was performed with ITK-SNAP software (version 3.8.0, http://www.itksnap.org (accessed on 20 January 2020)).

Radiomics features in the two types of ROIs among the four sequences of each patient were extracted with the open-source platform called PyRadiomics. The features extracted comprised 18 first-order features, 14 grey-level difference matrix (GLDM) features, 22 grey-level co-occurrence matrix (GLCM) features, 16 grey-level run length matrix (GLRLM) features, and 16 grey-level size zone matrix (GLSZM) features. For each patient, 86 features in 8 regions could be extracted for a total of 688 features. All features were normalized by the Z-score before feature screening. The radiomics features were extracted as an input in the deep learning model. The sequences were registered to the same physical space so that the same patient ROIs matched the same lesion area in each sequence. Following preprocessing, the patients were randomly divided into training and testing sets (80%/20% split).

### 2.4. ResNet Network Overview and Analysis

A convolutional neural network with a depth of 18 layers (ResNet-18) was selected as the prediction model. The training set was used to implement the deep learning model to predict the status of MGMT promoter methylation, and the validation set was used to evaluate the performance of the model. Radiomics features in two types of ROIs of the four sequences for each patient were separately or combinatorially used as an input. Five-fold cross-validation experiments were used to optimize this architecture. The input image should have a size of 224×224 pixels, so the radiomics features were treated as greyscale images and transformed to the required size as the input.

The final network architecture is shown in Figure S1. The softmax function was used in the last layer, and two 3×3 convolutional layers were contained in each residual unit. To determine the importance of each feature, we removed the final average pool and fully connected layers after the conv5 layer. Instead, the output with a size of 7 × 7 × 512 after the conv5 layer was connected to an upsample layer with an output size of 86 (the total number of features) × total number of modalities and ROIs × 512. (i.e., in the case of all four image maps and all ROIs used, the output size would be 86 × 8 × 512). The modified layer enabled us to gain the weight of each feature and rank their importance since the output size exactly matches the size of the input data. The upsampling layer was followed by a convolutional layer with an output size of 86 × 8 × 2, and the label was upsampled to a size of 86 × 8 × 1, with all elements inside the same layer as its original label (i.e., if the label is 1, then the label in the model is 1 of size 86 × 8 × 1).

Then, the predefined neural network model ResNet-18 was implemented by PyTorch, which is a framework used for neural networks in Python. The hyperparameters were the epochs (200, 400, and 600 epochs) and learning rates (0.1, 0.01, and 0.001). For each single MRI modality or combination of modalities, we tuned those hyperparameters and used cross-entropy loss to predict the binary classification. The model was trained with a momentum of 0.9, a weight decay of 0.0004, a batch size of 32, and stochastic gradient descent. The area under the curve (AUC) and average accuracy were used as the criteria for evaluation, and other metrics, such as sensitivity (SENS), specificity (SPEC), positive predictive value (PPV), negative predictive value (NPV), F1 score, and Matthew’s correlation coefficient (MCC), were also calculated. A brief explanation of the above-mentioned performance metrics is given in Supplementary Methods Section.

**Figure 3:**
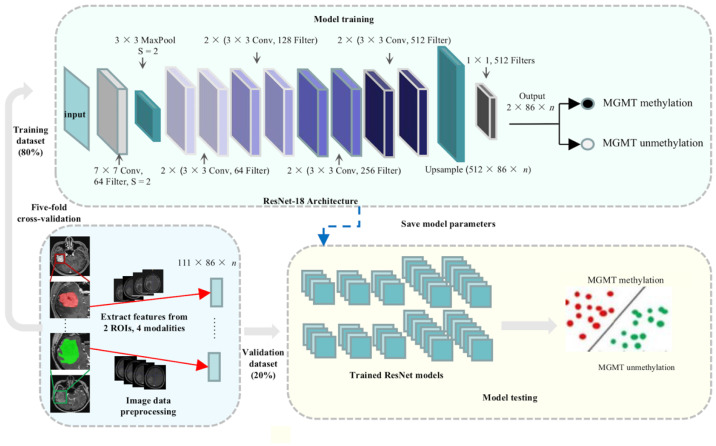
Structure of the deep learning model. Residual Networks extract features from 2 ROIs of 4 sequences were developed and evaluated with five-fold cross-validation, where in each fold, the dataset was randomly divided into training (80%) and validation (20%) cohorts.

We compared the accuracy metrics of the models to determine the best model and selected the 10 most important features of the best model via a class activation map. The structure of the deep learning model is shown in Figure 3.

### 2.5. Statistical Analysis

A statistical analysis of basic clinical information was performed using SPSS software (SPSS 23.0 statistical package; SPSS Inc., Armonk, NY, USA: IBM Corp.). A chi-squared test was performed to determine significant differences in sex and tumor type between the two groups. Differences in the age distribution were evaluated using Student’s t-test. A *p*-value < 0.05 was considered statistically significant.

## 3. Results

### 3.1. Patient Characteristics

A total of 117 patients were eligible for analysis, and 6 patients were excluded because of inadequate MR images (*n* = 4, patients did not have T2-weighted images) or poor image quality (*n* = 2). A total of 111 patients with complete MRI imaging data and complete results of MGMT promoter methylation status were enrolled in the training (89 patients) and validation cohorts (22 patients), including 56 patients with MGMT methylated gliomas and 55 patients with MGMT unmethylated gliomas. The characteristics of the patients in this study cohort are shown in Table 1. Significant differences in sex and age were not observed between the two groups.

### 3.2. ResNet Model Predictive Capacity Using a Single MRI Modality

Radiomics features in two types of ROIs among the four sequences of each patient were used as a ResNet input. The following results were achieved in the classification experiments. Based on the ROI of the whole tumor, including the parenchyma and edema, the predictive capacity of the T1CE model achieved the highest AUC value (0.81). Based on the ROI of the tumor core, the predictive capacity of the T1CE model achieved the highest AUC value (0.84).

### 3.3. ResNet Model Predictive Capacity Using Multiple MRI Sequences

Radiomics features in two types of ROIs among the four sequences of each patient were used in combination as an input. The following results were achieved in the classification experiments. Based on the ROI of the whole tumor, including the parenchyma and edema, the predictive capacity of the T1CE combined with the ADC model achieved the highest AUC value (0.85). Based on the ROI of the tumor core, the predictive capacity of the T1CE combined with the ADC model achieved the highest AUC value (0.90).

### 3.4. Model Comparison and the Final Model

**Figure 4:**
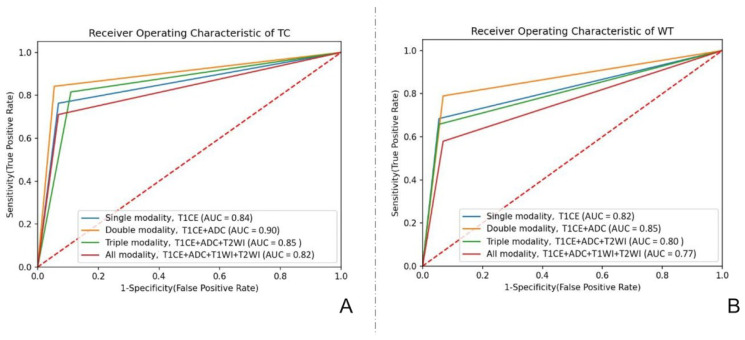
Receiver operating characteristic (ROC) curves based on 2 ROIs of different modal models with the highest AUC values. (**A**) ROC curves based on the ROI of the tumor core of different modal models with the highest AUC values. (**B**) ROC curves based on the ROI of the whole tumor of different modal models with the highest AUC values.

The results of all the models are shown in Table 2. Receiver operating characteristic (ROC) curves based on two ROIs of each modality, including single-, dual-, triple-, and full-modal models, with the highest AUC values are shown in Figure 4. According to the model comparison, the T1CE combined with the ADC model based on the ROI of the tumor core exhibited the best performance, with the highest accuracy (0.91) and AUC (0.90) among all the models. The importance of the features of the best model was calculated via a class activation map and is shown in Table S2.

The ten most important features were original GLDM Dependence Entropy, original GLDM Dependence NonUniformity, original GLDM Dependence NonUniformity Normalized, original GLDM Dependence Variance, original GLDM Grey Level NonUniformity, original GLDM Grey Level Variance, original GLDM High Grey Level Emphasis, original GLDM Large Dependence Emphasis, original GLDM Large Dependence High Grey Level Emphasis, and original GLDM Large Dependence Low Grey Level Emphasis.

## 4. Discussion

In this study, a ResNet based on radiomics features was proven to be a feasible tool for predicting the MGMT promoter methylation status of gliomas. Among single MRI modalities, the T1CE model based on the ROI of the tumor core achieved the highest AUC value (0.84). Among multiple MRI modalities, the T1CE combined with the ADC model based on the ROI of the tumor core achieved the highest AUC value (0.90). In the final model, the T1CE combined with the ADC model based on the ROI of the tumor core exhibited the best performance, with the highest accuracy (0.91) and AUC (0.90) among all the models. Ten features were selected as the most important radiomics features for the prediction. Our findings suggest that T1CE combined with ADC may be superior to other single or multiple MRI sequences in the prediction of MGMT promoter methylation. The model trained with T1CE with ADC achieved better results, followed by the model trained with T1CE images. Our study demonstrates that a deep learning model based on radiomics features could help to identify molecular biomarkers from routine medical images and further facilitate treatment planning.

The results of our study support similar studies that have shown that machine learning, especially deep learning, may be useful in the prediction of MGMT promoter methylation. In addition, the AUC of 0.90 achieved via T1CE based on the ROI of the tumor core is similar to the results from other studies that used radiomics analysis or deep learning-based MR image analysis. Tian et al. [20] suggested that the texture features extracted from T1CE might lead to the high performance of grading gliomas. Reza et al. [21] also reported that the radiomic features extracted from T1CE were more important than those extracted from other structural MRI sequence images in accordance with the results of feature importance ranking in the feature selection. Consistent with the findings of previous studies [22,23], our results indicated that the T1CE model based on the ROI of the tumor core performed better than other single models extracted from the other sequences. A possible reason for the performance was that features extracted from T1CE images contained more useful information. Unlike the above-mentioned studies, our findings suggest that the T1CE combined with the ADC model based on the ROI of the tumor core may be superior to the combination of all MRI sequences in the prediction of MGMT promoter methylation. A potential reason is the relatively poor imaging resolution of other sequences, which limited the stability and robustness of the derived radiomics features [24].

Instead of using images as a ResNet input, as in traditional research, our research extracted features from selected ROIs in different modalities from MR images and used them as the input for the model. Conventional radiomics studies use MR images as an input and adopt deep learning algorithms to train the model and evaluate the performance. However, features are extracted from all regions and modalities, and some regions may not be discriminative and may cause noise; therefore, we innovatively changed the feature extraction method. The features were treated as greyscale images in ResNet-18 [25], which is a residual neural network model used for image recognition.

Machine learning approaches allow the classification of individual genetic mutations of gliomas, and the prediction of MGMT promoter methylation was also realized through deep learning algorithms [26,27]. The ResNet-18 neural network is a convolutional neural network with a depth of 18 layers, which was proposed for image classification in 2015. A ResNet consists of residual blocks with the same data dimension in the early stage, while in the later stage, the data are sampled downward, and the number of layers increases. Deeper architectures based on ResNet are reported to yield better results [28]. Our study revealed the value of radiomics features in predicting MGMT methylation status. Radiomics has already been widely adopted for the noninvasive analysis of genetic and clinical information in different medical fields. Radiomics can be used to obtain information by extracting a great number of features from lesion areas. The features used for different diseases are similar, including intensity, shape, texture, wavelet, and other descriptive features [29]. Several similar attributes have previously been reported, such as texture features in the whole tumor, and studies have specifically found that a GLSZM of low grey-level emphasis could delineate the MGMT status with an AUC of 0.71 [30]. Texture features extracted from contrast-enhanced T1WI adequately separated IDH-mutant gliomas from IDH wild-type gliomas [31]. Consistent with the above-mentioned research, our study found that the 10 most important radiomics features were GLDM features, a category of texture features.

Our study has some general limitations. First, we recruited only 111 patients in this study. Although radiomics can be performed with as few as 100 patients, recruiting more patients will provide more power and better support our findings in the future. Second, we did not include clinical data nor functional MR imaging data, such as MR spectroscopy and dynamic contrast-enhanced MRI, which may add more value to the prediction model. Third, an independent external cohort that met the inclusion and exclusion criteria from other hospitals was requested to validate the model and address overfitting.

## 5. Conclusions

In summary, deep learning based on radiomics features is able to noninvasively predict the MGMT promoter methylation status in patients with diffuse gliomas with a high accuracy. The model trained with T1CE with ADC achieved the best results, followed by the model trained with T1CE images. This prediction model may aid in providing more precise diagnoses and guiding treatment decisions. Further efforts are needed to explore the full value of deep learning based on radiomics using functional MR imaging data and clinical data to establish an optimal model for routine clinical use.

## Figures and Tables

**Table 1:** Characteristics of the two groups.

Group Parameters	Total	MGMT Methylation	MGMT Unmethylation	*p*-Value
Sex (males/females, No.)	111	26/30	36/19	0.056 ^a^
Age (mean ± SD, years)	-	53.45 ± 13.61	55.85 ± 13.18	0.346 ^b^
Glioblastoma	65 (58.6%)	25	40	0.002 ^a^
Anaplastic astrocytoma	6 (5.4%)	2	4	
Diffuse astrocytoma	20 (18.4%)	13	7	
Anaplastic oligodendrocytoma	9 (8.1%)	5	4	
Oligodendrocytoma	11 (9.9%)	11	0	

Abbreviations: oxygen 6-methylguanine-DNA methyltransferase (MGMT). Notes: ^a^: chi-squared test; ^b^: Student’s *t*-test. Unless otherwise noted, the data in the table refer to the number of patients, with percentages in parentheses.

**Table 2:** Results of all models.

ROI	Modality	Model	AUC	ACC	F1 Score	SENS	SPEC	PPV	NPV	MCC
WT	single	T1CE	0.82	0.86	0.83	0.69	0.95	0.88	0.68	0.68
		ADC	0.71	0.79	0.74	0.48	0.94	0.79	0.49	0.49
		T1WI	0.75	0.78	0.75	0.63	0.88	0.70	0.51	0.51
		T2WI	0.68	0.78	0.71	0.41	0.94	0.79	0.40	0.40
	double	ADC + T1CE	0.85	0.88	0.86	0.76	0.93	0.86	0.72	0.72
		T1WI + ADC	0.76	0.79	0.75	0.63	0.89	0.79	0.56	0.56
		T1WI + T1CE	0.82	0.84	0.82	0.75	0.89	0.77	0.64	0.64
		T1WI + T2WI	0.69	0.77	0.71	0.43	0.95	0.81	0.46	0.46
		T2WI + ADC	0.70	0.78	0.73	0.49	0.92	0.77	0.43	0.43
		T2WI + T1CE	0.81	0.85	0.82	0.69	0.93	0.84	0.66	0.66
	triple	T1CE + ADC + T1WI	0.78	0.83	0.80	0.66	0.90	0.77	0.59	0.59
		T1WI + T2WI + ADC	0.71	0.77	0.72	0.52	0.91	0.76	0.47	0.47
		T1WI + T2WI + T1CE	0.78	0.81	0.79	0.65	0.91	0.76	0.57	0.57
		T2WI + ADC + T1CE	0.80	0.85	0.82	0.65	0.94	0.88	0.65	0.65
	all	T1WI + T2WI + T1CE + ADC	0.77	0.81	0.78	0.60	0.93	0.84	0.58	0.58
TC	single	T1CE	0.84	0.87	0.85	0.75	0.93	0.84	0.70	0.70
		ADC	0.73	0.79	0.76	0.50	0.95	0.87	0.53	0.53
		T1WI	0.51	0.73	0.66	0.31	0.71	0.53	0.24	0.24
		T2WI	0.76	0.80	0.76	0.66	0.86	0.71	0.54	0.54
	double	ADC + T1CE	0.90	0.91	0.90	0.86	0.95	0.89	0.81	0.81
		T1WI + ADC	0.69	0.77	0.71	0.49	0.90	0.78	0.45	0.45
		T1WI + T1CE	0.86	0.89	0.87	0.78	0.94	0.89	0.75	0.75
		T1WI + T2WI	0.72	0.79	0.75	0.50	0.93	0.81	0.50	0.50
		T2WI + ADC	0.67	0.76	0.72	0.43	0.90	0.73	0.37	0.37
		T2WI + T1CE	0.81	0.86	0.84	0.70	0.91	0.85	0.65	0.65
	triple	T1CE + ADC + T1WI	0.85	0.87	0.85	0.81	0.88	0.81	0.70	0.70
		T1WI + T2WI + ADC	0.72	0.78	0.73	0.53	0.90	0.75	0.48	0.48
		T1WI + T2WI + T1CE	0.81	0.86	0.83	0.69	0.94	0.90	0.68	0.68
		T2WI + ADC + T1CE	0.83	0.86	0.83	0.74	0.91	0.84	0.68	0.68
	all	T1WI + T2WI + T1CE + ADC	0.82	0.86	0.83	0.72	0.93	0.85	0.68	0.68

Abbreviations: area under the curve (AUC); average accuracy (ACC); sensitivity (SENS); specificity (SPEC); positive predictive value (PPV); negative predictive value (NPV); Matthew’s correlation coefficient (MCC). Notes: WT means the ROI of the whole tumor, including the parenchyma and edema. TC means the ROI of the tumor core.

## Data Availability

The data that support the findings of this study are available from the corresponding author, Xin Zhang, upon reasonable request.

## Supplementary Materials

The following supporting information can be downloaded at: https://www.mdpi.com/article/10.3390/jcm11123445/s1, Table S1. MRI protocol, Table S2. Importance of features of the best model, Figure S1. Residual network architecture, supplementary methods section.
