# Supplementary material for: Predicting MGMT Promoter Methylation in Diffuse Gliomas Using Deep Learning with Radiomics
Source: J Clin Med. 2022 Jun 15;11(12):3445. doi: 10.3390/jcm11123445 (PMC9224690; doi:10.3390/jcm11123445)
Supplement: Supplementary file 1 [file jcm-11-03445-s001.zip › jcm-1722872-supplementary.pdf]

Supplementary materials

Table S1. MRI protocol.

Instrument	Sequence	Slice (mm)	Voxel size (mm * mm * mm)	FOV (mm * mm)	TR (ms)	TE (ms)	Bandwidth
uMR770 (3.0 T)	DWI	6	1.67 * 1.67 * 6.00	240 * 230	1800	86	1790
	T2WI	1	1 * 1 * 1	256 * 272	1500	369.6	520
	T1WI	6	1.02 * 0.76 * 6.00	220 * 220	230	4.49	260
	T1CE	1	1.00 * 1.00 * 1.00	256 * 270	7.2	3.1	250
Achieva (3.0 T)	T2WI	6	0.55 * 0.68 * 6	230 * 184	2223	80	168.4
	DWI	6	1.5 * 1.89 * 6	230 * 230	2359	89	20
	T1WI	6	0.9 * 1.12 * 6	230 * 183	250	2.3	173.1
	T1CE	6	0.65 * 0.82 * 6	230 * 183	230	2.3	216.6

Abbreviations: T1 weighted image (T1WI), T2 weighted image (T2WI), diffusion-weighted imaging (DWI), T1 contrast-enhanced (T1CE), repetition time (TR), echo time (TE), field of view (FOV)

Table S2. Importance of features of the best model

Feature	importance
original_gldm_DependenceEntropy	4292.799
original_gldm_DependenceNonUniformity	4292.799
original_gldm_DependenceNonUniformityNormalized	4292.799
original_gldm_DependenceVariance	4292.799
original_gldm_GrayLevelNonUniformity	4292.799
original_gldm_GrayLevelVariance	4292.799
original_gldm_HighGrayLevelEmphasis	4292.799
original_gldm_LargeDependenceEmphasis	4292.799
original_gldm_LargeDependenceHighGrayLevelEmphasis	4292.799
original_gldm_LargeDependenceLowGrayLevelEmphasis	4292.799
original_gldm_LowGrayLevelEmphasis	4292.799
original_gldm_SmallDependenceEmphasis	4292.799
original_gldm_SmallDependenceHighGrayLevelEmphasis	4292.799
original_gldm_SmallDependenceLowGrayLevelEmphasis	4292.799
original_glszm_ZoneVariance	4291.2104
original_glszm_ZonePercentage	4281.681
original_glszm_ZoneEntropy	4272.151

original_glszm_SmallAreaLowGrayLevelEmphasis	4262.62
original_glszm_SmallAreaHighGrayLevelEmphasis	4253.0903
original_glszm_SmallAreaEmphasis	4243.56
original_glszm_SizeZoneNonUniformityNormalized	4234.0293
original_glszm_SizeZoneNonUniformity	4224.4995
original_glszm_LowGrayLevelZoneEmphasis	4214.969
original_glszm_LargeAreaLowGrayLevelEmphasis	4205.4385
original_glszm_LargeAreaHighGrayLevelEmphasis	4195.9087
original_glszm_LargeAreaEmphasis	4186.378
original_glszm_HighGrayLevelZoneEmphasis	4176.8477
original_glszm_GrayLevelVariance	4167.3184
original_glszm_GrayLevelNonUniformityNormalized	4157.788
original_glszm_GrayLevelNonUniformity	4148.2573
original_glrlm_ShortRunLowGrayLevelEmphasis	4138.7275
original_glrlm_ShortRunHighGrayLevelEmphasis	4129.197
original_glrlm_ShortRunEmphasis	4119.667
original_glrlm_RunVariance	4110.136
original_glrlm_RunPercentage	4100.6055
original_glrlm_RunLengthNonUniformityNormalized	4091.076
original_glrlm_RunLengthNonUniformity	4081.5457
original_glrlm_RunEntropy	4072.0151
original_glrlm_LowGrayLevelRunEmphasis	4062.4854
original_glrlm_LongRunLowGrayLevelEmphasis	4052.955
original_glrlm_LongRunHighGrayLevelEmphasis	4043.425
original_glrlm_LongRunEmphasis	4033.8945
original_glrlm_HighGrayLevelRunEmphasis	4024.364
original_glrlm_GrayLevelVariance	4017.4102
original_glrlm_GrayLevelNonUniformityNormalized	4013.0312
original_glrlm_GrayLevelNonUniformity	4008.6516
original_glcm_SumSquares	4004.273
original_glcm_SumEntropy	3999.8943
original_glcm_MaximumProbability	3995.5156
original_glcm_InverseVariance	3991.1362
original_glcm_Idn	3986.7578
original_glcm_Id	3982.379
original_glcm_Idmn	3978.0002
original_glcm_Idm	3973.621
original_glcm_Imc2	3969.242
original_glcm_Imc1	3964.8638
original_glcm_JointEntropy	3960.4846
original_glcm_JointEnergy	3956.1057
original_glcm_DifferenceVariance	3951.727
original_glcm_DifferenceEntropy	3947.3481
original_glcm_DifferenceAverage	3942.969
original_glcm_Correlation	3938.5906
original_glcm_Contrast	3934.2112

original_glcm_ClusterTendency	3929.833
original_glcm_ClusterShade	3925.454
original_glcm_ClusterProminence	3921.075
original_glcm_JointAverage	3916.6958
original_glcm_Autocorrelation	3912.317
original_firstorder_Variance	3907.9385
original_firstorder_Uniformity	3903.5596
original_firstorder_TotalEnergy	3899.1807
original_firstorder_Skewness	3894.8015
original_firstorder_10Percentile	3894.072
original_firstorder_90Percentile	3894.072
original_firstorder_Energy	3894.072
original_firstorder_Entropy	3894.072
original_firstorder_InterquartileRange	3894.072
original_firstorder_Kurtosis	3894.072
original_firstorder_Maximum	3894.072
original_firstorder_MeanAbsoluteDeviation	3894.072
original_firstorder_Mean	3894.072
original_firstorder_Median	3894.072
original_firstorder_Minimum	3894.072
original_firstorder_Range	3894.072
original_firstorder_RobustMeanAbsoluteDeviation	3894.072
original_firstorder_RootMeanSquared	3894.072

Notes: The numerical value listed in S2 is to visualize the contribution and response of features to the model. the values reflect the importance of features. The larger the value is, the more important the feature is. The values have been calculated via class activation maps (CAMs), which can serve as a quality assurance tool such that they highlight image regions relevant to the model's prediction and denote the model's confidence in the prediction.

Layer Name	Output Size	ResNet-18
conv1	$112 \times 112 \times 64$	7×7, 64, stride 2
conv2_x	$56 \times 56 \times 64$	3×3 max pool, stride 2
		$\begin{bmatrix} 3 \times 3, 64 \\ 3 \times 3, 64 \end{bmatrix} \times 2$
conv3_x	$28 \times 28 \times 128$	$\begin{bmatrix} 3 \times 3, 128 \\ 3 \times 3, 128 \end{bmatrix} \times 2$
conv4_x	$14 \times 14 \times 256$	$\begin{bmatrix} 3 \times 3, 256 \\ 3 \times 3, 256 \end{bmatrix} \times 2$
conv5_x	$7 \times 7 \times 512$	$\begin{bmatrix} 3 \times 3, 512 \\ 3 \times 3, 512 \end{bmatrix} \times 2$
average pool	$1 \times 1 \times 512$	7×7 average pool
fully connected	1000	512×1000 fully connections
softmax	1000	

Figure S1. Residual Network architecture

Supplementary methods

Evaluation metrics for prediction results.

1) Classification accuracy

The percentage of predicted values that exactly match with actual values. As five-fold cross validation was used, the average value of the accuracy score in five folds is calculated and used for model selection.

$$Accuracy_i = \frac{No.\ of\ correct\ predictions_i}{Total\ No.\ of\ predictions_i} = \frac{TP+TN}{(TP+FN)+(FP+TN)}$$

$$\overline{Accuracy} = \frac{1}{5}\sum_{i=1}^{5} Accuracy_i$$

Where $Accuracy_i$ is the accuracy score of the ith fold, and $\overline{Accuracy}$ is the average accuracy of five folds.

2) F1 Score

F1 Score is a weighted average of the precision and recall where the precision is the number of true positive results divided by the number of all positive results, and the recall is the number of true positive results divided by the number of all samples that should have been identified as positive. F1 Score is a better metric when the dataset is imbalanced and biased.

$$F1\ Score = 2 \times \frac{Precision \times Recall}{Precision + Recall} = \frac{2 \times TP}{2 \times TP + FP + FN}$$

3) Other metrics:

$$\text{AUC} = \text{area under receiver operating characteristic curve}$$

$$\text{Sensitivity (SENS)} = \frac{\text{TP}}{\text{TP} + \text{FN}}$$

$$\text{Specificity (SPEC)} = \frac{\text{TN}}{\text{FP} + \text{TN}}$$

$$\text{Positive Predictive Value (PPV)} = \frac{TP}{TP + FP}$$

$$\text{Negative Predictive Value (NPV)} = \frac{TN}{TN + FN}$$

$$\text{Mathew's Correlation Coefficient (MCC)} = \frac{\text{TP} \times \text{TN} - \text{FP} \times \text{FN}}{\sqrt{(\text{TP} + \text{FP})(\text{TP} + \text{FN})(\text{TN} + \text{FP})(\text{TN} + \text{FN})}}$$

where TP, FP, TN and FN represent the regions of the true positive, the false positive, true negative and the false negative, respectively.
